# Evaluation of Subcision for the Correction of the Prominent Nasolabial Folds

**DOI:** 10.1155/2015/976153

**Published:** 2015-12-16

**Authors:** R. M. Robati, F. Abdollahimajd, A. M. Robati

**Affiliations:** ^1^Skin Research Center, Shahid Beheshti University of Medical Sciences, Shohada-e Tajrish Hospital, Shahrdari Sreet, Tehran 1989934148, Iran; ^2^Department of Surgery, Bam University of Medical Sciences, Bam, Iran

## Abstract

*Background.* A prominent nasolabial fold (NLF) is a cosmetic problem. Currently, numerous therapeutic modalities are available for pronounced NLFs with variable efficacy.* Objective.* To determine the efficacy and safety of subcision using a hypodermic needle for the correction of the prominent NLFs and its effect on skin elasticity.* Methods.* Sixteen patients with prominent NLFs underwent subcision. The investigators' assessment of improvement and the patients' satisfaction were both recorded 1 and 6 months after the procedure. Also, we evaluate the skin elasticity of NLFs before and after the treatment using a sensitive biometrologic device with the measurement of cutaneous resonance running time (CRRT).* Results.* Thirteen (81.25%) patients showed a moderate improvement at 1st month and 13 (81.25%) patients had at least a mild improvement at 6th month. There was no persistent side effect lasting more than a few days. Mean CRRT at 1 and 6 months after the treatment was significantly higher compared to the baseline.* Conclusion.* Subcision may be considered effective for the correction of pronounced NLFs. However, further controlled studies with larger sample size are necessary to assess the efficacy of this technique in particular with use of more objective assessment of skin biometric characteristics. This trial is registered with IRCT201108097270N1 (registered on January 27, 2012).

## 1. Introduction

The nasolabial fold (NLF) is a unique boundary between the cheek, mouth, and chin [1, 2]. Its shape can be concave, convex, or straight. It is generally imperceptible in children, in part, due to their skin elasticity [1]. The NLF often becomes apparent around the age of 25 years and then becomes more obvious with aging as a combination of thinning and relaxation of the skin [3, 4]; also other factors can be effective on this process such as ultraviolet radiation [1]. A prominent nasolabial crease is a cosmetic problem. Currently, numerous therapeutic options such as synthetic dermal fillers [1], fat grafts [4], Nd:YAG laser [5], radiofrequency device [6], direct excision or rhytidectomy [7, 8], and intense focused ultrasound [9] are available for improvement of a pronounced NLF with variable outcomes.

Subcision or subcutaneous incisionless surgery was first described by D. S. Orentreich and N. Orentreich in 1995. It is a surgical modality for the treatment of wrinkles and depressed scars with a tribeveled hypodermic needle inserted under the depressed area [10]. The releasing action of this procedure separates the fibrotic attachment. Moreover, the connective tissue forms during the wound-healing process, so the depression is raised [11]. Subsequently, several reports introduced that subcision with various types of needles or blades is effective for wrinkles, depressed scars, and prominent NLFs, as well as for acne scars [12–16]. The NLF is often a function of dermal atrophy. Therefore, subcision may be a useful option for the treatment of NLF not only due to its releasing action but also due to its induction of connective tissue formation.

In general, few studies have assessed the efficacy and safety of subcision in the treatment of prominent NLFs. In this study, we assess the efficacy and safety of subcision for the correction of pronounced NLFs. Also, we quantitatively evaluate the efficacy of this technique on skin elasticity using a sensitive biometrologic device (Reviscometer RVM 600) before and after the treatment. Reviscometer RVM 600 is useful in evaluating the skin elasticity and the direction of skin tension lines based on resonance running time [17].

## 2. Patients and Methods

### 2.1. Patients

This study is an open-label clinical trial. Sixteen patients with prominent NLFs were recruited to the study; all of them were female in the age group from 33 to 60 years. This study was approved by the ethical committee of Shahid Beheshti University of Medical Sciences and was performed according to the principles of the Declaration of Helsinki. All of the subjects signed a written informed consent after explanation of the procedure. Exclusion criteria included those who had connective tissue disorders (acquired or inherited), had a bleeding diathesis or a history of coagulation disorders in which anticoagulation therapies or medications such as aspirin and vitamin E prolonged the bleeding time, had susceptibility to keloid formation, were of age less than 30 years, were pregnant or lactating females, had active infection or history of cutaneous malignancy on the face, were on topical treatment (except for sunscreen agents) for the last 4 weeks, had isotretinoin therapy in the last 12 months, and had inability to attend follow-up visits. Cosmetic procedures such as laser or neurotoxin injection anywhere on the face in 12 months and filler injection anywhere on the face in 5 years before the study were also regarded as exclusion criteria.

### 2.2. Methods

Baseline demographic data of all patients were recorded at the start of the study. Photographs of the affected anatomic sites were taken before subcision as well as 1 and 6 months after the treatment with the same digital camera. The grade of wrinkles (in NLF) was evaluated by a wrinkle severity rating scale (WSRS) [18] before the treatment and at 1 and 6 months after the treatment as follows:

 1 = absent (no visible fold), 2 = mild (shallow fold), 3 = moderate (not visible when stretched), 4 = severe (prominent, long, and deep fold), and 5 = extreme (extremely deep and long folds detrimental to facial appearance).

Outcomes and side effects of subcision procedure were evaluated by investigators at 1 and 6 months after the treatment. Moreover, another dermatologist who was blinded to the clinical data performed these evaluations. The degree of improvement was scored as follows:

 No response is <10%; mild response is 10–25%; moderate response is 26–75%; and excellent response is >75%.

The patients were asked to complete a questionnaire to determine their satisfaction (no, relative, or absolute satisfaction) and treatment complications (bruising, hematoma, hemorrhage, and keloid) during each follow-up visit.

### 2.3. Skin Elasticity

**Figure 1:**
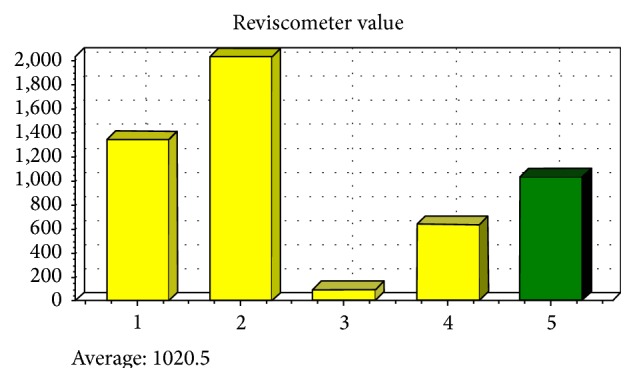
The diagrams provided by Reviscometer RVM 600 probe after measurement of CRRT.

The loss of elasticity is one of the important characteristics of skin aging. Reviscometer RVM 600 is a sensitive biometrologic device used to measure the skin elasticity. The measurement is based on cutaneous resonance running time (CRRT) of an acoustical shockwave. It determines the mechanical properties of the skin and the direction of collagen and elastic fibers. The CRRT is expressed in arbitrary units (AU) [19]. Two sensors are applied to the skin surface in supine position. The mean CRRT over the four axes (0°, 180°, 90°, and 270°) was calculated for the NLFs. These measurements were conducted at room temperature 24–26°C with a relative humidity of 50 ± 3%. These measurements were recorded for each patient before the treatment and also repeated at each visit (Figure 1).

### 2.4. Subcision Procedure

The procedures were performed by the same dermatologist under constant conditions using a substantially identical method. The subcision was performed with the use of an 18-gauge hypodermic needle (Nokor Admix, Becton Dickinson Co.). First, the subcutaneous anesthesia (lidocaine 1% without epinephrine) was performed and then the needle was inserted, with blade facing upward, through a tiny incision in the middle of each NLF. Afterward, it was turned so that the tip was in a horizontal orientation (parallel to skin surface) just below the dermal-subcutaneous junction plane and a gentle fanning motion (side-to-side needle motion) was performed. The needle passed along these folds parallel to the skin surface. In addition, a gentle piston-like motion was used to advance the needle just below the dermal-subcutaneous junction plane. The width of the subcised area was approximately 7 mm around each NLF. We performed the procedure until the fold was effaced. We did subcision in upward direction to the proximal NLF. Afterward, the needle was inserted downward into the previous entry site and the mentioned procedure was repeated for distal portion of the NLF. We performed subcision just below the dermal-subcutaneous junction plane with no vigorous motion avoiding deeper structure to protect vessels especially facial artery branches. After the procedure, a piece of gauze without pressure was applied on the entry site for 24 h; also topical and oral antibiotics were prescribed.

### 2.5. Statistical Analysis

All descriptive statistics are summarized as mean + SD or frequency (%). Comparison of outcome variables between any two time points was done by Wilcoxon Signed Ranks Test. Trend of measures for grade of wrinkles as an ordinal variable was investigated using Friedman's test statistic. For skin elasticity, the trend of measurement was assessed by repeated measures ANOVA test.

## 3. Results

### 3.1. Baseline Data

Sixteen female patients were included in this study. Mean age of patients was 45.1 years (SD = 9.8). Most of the patients (62.5%) had a moderate grade of wrinkles at the beginning of the study followed by four patients in the severe grade, one in mild grade, and one in extreme grade. Mean skin CRRT was 1007.5 ± 494.2 (AU) before the treatment. Baseline demographics and outcomes of the subcision procedure are presented in Table 1.

### 3.2. After Treatment

**Figure 2:**
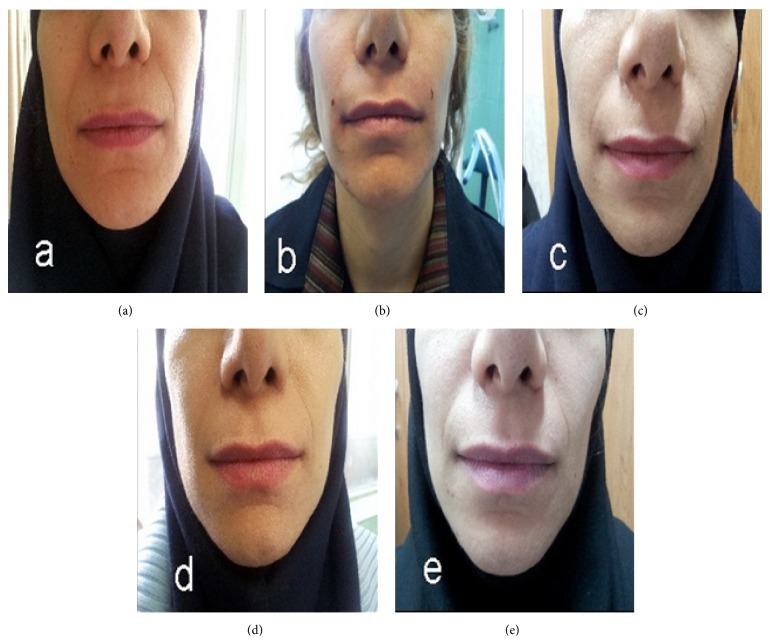
The considerable improvement of nasolabial folds after subcision; (a) before and (b) immediately after treatment (>75% improvement), (c) one month (50–75% improvement) and (d) three months after subcision (25–50% improvement), and (e) six months after treatment (≈25% improvement especially in upper portion of NLFs). No depression at the needle insertion points was seen.

**Figure 3:**
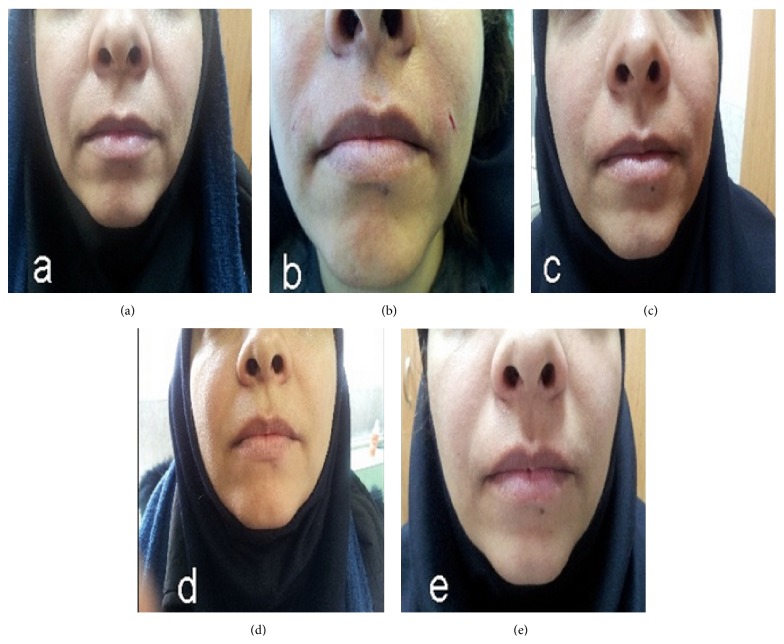
Marked improvement of nasolabial folds after subcision: (a) before and (b) immediately after treatment (>75% improvement), (c) one month (50–75% improvement) and (d) three months after subcision (25–50% improvement), and (e) six months after procedure (≈25% improvement). No depression at the needle insertion points was seen.

The grades of improvement in WSRS (compared to baseline) in different time points of the study are shown in Table 2. Comparison of the grade of wrinkles at 1 and 6 months after the treatment with baseline data showed a significant difference (1 month versus baseline: test statistic = −3.69, *p* < 0.001; 6 months versus baseline: test statistic = −3.00, *p* = 0.003). Trend of measurement in different time points was also statistically significant (Friedman's test statistics = 23.5, *p* < 0.001). Mean degree of improvement was 42.8 ± 18.7% at 1 month and 18.8 ± 10.8% at 6 months after the treatment. The improvement degrees (compared to baseline) at one and 6 months after procedure are shown in Table 3. Patients showed less improvement after 6 months compared to 1 month after the treatment (Wilcoxon Signed Ranks Test statistic: −3.05, *p* = 0.002) (Figures 2 and 3).

One month after the treatment, patients had higher satisfaction. We found that 4 (25%) patients had moderate satisfaction and 12 (75%) patients were fully satisfied. Six months after the treatment, 3 (18.75%) patients had no satisfaction, 9 (56.25%) patients had moderate satisfaction, and 4 (25%) patients were fully satisfied. Satisfaction after 6 months was significantly lower than 1 month after the intervention (Wilcoxon Signed Ranks Test statistic: −3.05, *p* = 0.002). Also, 5 (31.25%) patients had only mild bruising that resolved after 2-3 days in all of them. One and 6 months after the treatment, there was no complication.

Mean CRRT was 1007.5 ± 494.2 (AU) before the treatment. It was 1369.1 ± 582.9 (AU) and 1359.9 ± 622.9 (AU) 1 and six months after the treatment, respectively. Comparison of mean CRRT at 1 and 6 months after the treatment to baseline data showed a significant difference (1 month versus baseline: *t*-test statistic = −2.46, *p* = 0.02; 6 months versus baseline: *t*-test statistic = −2.42, *p* = 0.03). Trend of measurement in different time points was also statistically significant (repeated measures test statistics = 1239408, *p* = 0.02).

## 4. Discussion

The treatment plan for prominent NLFs should be individualized according to some factors such as aesthetic needs, the patient's age, and economic burden [20]. Several modified subcision techniques were introduced for depressed scars and wrinkles and also for prominent NLFs [3, 12–15]. In our study, 13 (81.25%) patients showed moderate improvement at 1st month and 13 (81.25%) patients had at least mild improvement at 6th month. One month after the treatment, 75% of patients were fully satisfied and 6 months after the treatment, 81.25% of them had at least moderate satisfaction. There was no persistent side effect lasting more than a few days. No contracture in lower portion of NLF was observed.

The CRRT is influenced by collagen fibers in the upper dermis. As aging progresses, defragmentation of elastin network occurs and configuration of dermal collagen network changes, which could increase skin stiffness and decrease skin elasticity and CRRT [21]. In our study, mean CRRT at 1 and 6 months after the procedure was significantly higher compared to the baseline. The releasing action of this procedure separates the fibrotic attachments. Moreover, the new connective tissue with new elastin and collagen networks forms during the wound-healing process, so the depression is raised [11] and CRRT increased.

The immediate improvement after subcision seems to be the result of edema or mild bleeding under the NLFs. However, we followed the participants 1 and 6 months after the procedure to assess the persistence of improvement. The continued improvement may be due to the connective tissue formation after subcision. It would be better if we could perform a biopsy of NLF 6 months after the procedure to assess the histopathological pattern of possible neocollagenesis. Unfortunately, none of the participants agreed to undergo skin biopsy of NLFs after the procedure in this study.

To achieve the best results, it may be important to induce the formation of adequate amounts of scar tissue in the subcision area [3]. Although this is usually not complete with a single procedure, we can repeat this procedure at regular intervals to achieve more favorable and persistent results [3]. Moreover, it might be better to perform this study in a split-face design and do subcision only for right or left NLF to achieve a comparison of its efficacy. But the ethic committee of our center did not allow us to perform this pilot study in a split-face design with no treatment on a NLF. Unfortunately, we faced some restrictions of facilities to perform filler injection or fat graft for one side and subcision for the other side to compare them in a more controlled manner.

In conclusion, our study shows that subcision is an easy, safe, and inexpensive technique, with minimal complication and considerable rate of improvement for correction of prominent NSFs. It improves both the clinical appearance and skin elasticity of NLFs. However, we planned this study as a preliminary assay to evaluate the possible efficacy of subcision on the prominent NFLs. Therefore, we entered limited participants in this study with limited follow-up periods. So, further controlled studies with larger sample size and longer follow-up and use of more objective assessment of skin biometric or histopathologic characteristics are beneficial to elucidate the efficacy of subcision in improving NLFs more definitely.

## Figures and Tables

**Table 1:** Baseline demographics and outcomes of the subcision procedure in patients.

*N*	Sex/age	Dx. duration (year)	WSRS (0)	WSRS 1 m	WSRS 6 m	CRRT 0	CRRT 1 m	CRRT 6 m	Complication 1 wk	Complication 1, 6 m	Satis. 1 m	Satis. 6 m	Improve. % 1 m	Improve. degree 1 m	Improve. % 6 m	Improve. degree 6 m
1	f/42	10	3	2	3	1328	1835.2	1799	Bruising	No	Full	Relative	20	2	10	2
2	f/56	25	4	3	3	1427	1983	1891	Bruising	No	Full	Relative	70	3	30	3
3	f/59	30	4	3	3	460	657.25	623.2	No	No	Full	Relative	60	3	30	3
4	f/38	10	3	2	2	589	949	846.5	No	No	Full	Relative	40	3	20	2
5	f/33	2	3	2	3	920	950	895.5	No	No	Relative	No	20	2	5	1
6	f/36	3	2	1	2	2141.2	2200	2481.5	Bruising	No	Full	Full	50	3	20	2
7	f/60	25	5	3	4	949.3	958.75	952.25	No	No	Full	Relative	40	3	20	2
8	f/35	6	3	2	3	1406.8	1561.8	1410	No	No	Relative	Relative	50	3	20	2
9	f/52	20	4	3	3	560	890	818	Bruising	No	Full	Relative	50	3	30	3
10	f/36	6	3	2	2	194.2	2677	2594	No	No	Full	Full	30	3	15	2
11	f/35	5	3	2	3	1206	1430	1291	No	No	Full	No	25	3	5	1
12	f/60	34	4	3	4	460	589	480	Bruising	No	Relative	Relative	30	3	10	2
13	f/51	20	3	2	2	949	1251	1631	No	No	Full	Full	70	3	40	3
14	f/42	10	3	2	2	1305	1480	1447	No	No	Full	Relative	40	3	10	2
15	f/41	5	3	1	2	1376	1510	1626.2	No	No	Full	Full	70	3	30	3
16	f/46	8	3	3	3	849	983.75	972.5	No	No	Relative	No	20	2	5	1

*N*, number; Dx., disease; WSRS (0), Wrinkle Severity Rating Scale (base); m, month; CRRT, cutaneous resonance running time; wk, week; satis., satisfaction; improve., improvement.

*WSRS*: 1 = absent (no visible fold), 2 = mild (shallow fold), 3 = moderate (not visible when stretched), 4 = severe (prominent, long, and deep fold), and 5 = extreme (extremely deep and long folds detrimental to facial appearance).

*Improvement degree (evaluated by investigators and also one dermatologist)*: 1 = no response: <10%; 2 = mild response: 10–25%; 3 = moderate response: 26–75%; and 4 = excellent response: >75%.

**Table 2:** The grades of improvement in WSRS (compared to baseline) at one month and 6 months after the beginning of the procedure.

WSRS improvement	After 1 month	After 6 months
(compare to baseline)		
No change	1 (6.25%)	7 (43.75%)
At least 1 grade	15 (93.75%)	9 (56.25%)
2 grades or greater	2 (12.5%)	0

WSRS, Wrinkle Severity Rating Scale.

*WSRS*: 1 = absent (no visible fold), 2 = mild (shallow fold), 3 = moderate (not visible when stretched), 4 = severe (prominent, long, and deep fold), and 5 = extreme (extremely deep and long folds detrimental to facial appearance).

**Table 3:** Improvement degree (compared to baseline) at one month and 6 months after the beginning of the procedure.

Improvement degree	After 1 month	After 6 months
(compared to baseline)		
No response	0 (0)	3 (18.75%)
At least a mild response	3 (18.75%)	8 (50%)
A moderate response or greater	13 (81.25%)	5 (31.25%)

*Improvement degree (evaluated by investigators and also one dermatologist)*: 1 = no response: <10%; 2 = mild response: 10–25%; 3 = moderate response: 26–75%; and 4 = excellent response: >75%.
